# Case Report: Metastatic renal giant-cell sarcoma in an African pygmy hedgehog (*Atelerix albiventris*)

**DOI:** 10.3389/fvets.2025.1609488

**Published:** 2025-10-16

**Authors:** Pablo Díaz-Santana, Javier Déniz-Marrero, Cecilia Gola, Alejandro Suárez-Bonnet

**Affiliations:** ^1^Veterinary Pathology Centre, University of Surrey, Guildford, United Kingdom; ^2^Department of Pathobiology and Population Sciences, The Royal Veterinary College, Hatfield, United Kingdom

**Keywords:** immunohistochemistry, hedgehog, kidney, metastatic, sarcoma

## Abstract

The scientific literature referring to tumors in African pygmy hedgehog (*Atelerix albiventris*) is expanding, enhanced by an increased lifespan under human care. This study compiles the clinical records and pathologic findings in a 2-year and 7-month-old male African pygmy hedgehog (*Atelerix albiventris*) with a metastatic giant-cell renal sarcoma. Metastases to the lungs, retroperitoneum, lymph nodes, pericardium, and mediastinal adipose tissue were observed. Immunohistochemical assessment with a panel of 11 antibodies (cytokeratins [AE1/AE3, CK19, and CK20], vimentin, thyroid transcription factor-1, thyroglobulin, Ki67, Iba-1, S100, synaptophysin, and CD31) was performed, yielding negative immunolabeling in the neoplastic cells. To the author’s knowledge, neoplasms arising from the kidney in African pygmy hedgehogs are rarely reported, and a metastatic giant-cell renal sarcoma has not been previously described. This case report aims to contribute to the growing body of knowledge regarding neoplasm development and help veterinary specialists with the characterization of the pathologic conditions affecting this species.

## Introduction

The African pygmy hedgehog (*Atelerix albiventris*; Family Erinaceidae), or four-toed hedgehog, is an insectivorous and nocturnal small mammal gaining popularity as an exotic pet species in Western countries (1). Increasing literature has revealed a high incidence of malignant neoplastic events in captive individuals as they reach adulthood and geriatric ages (2, 3). The majority of the reported neoplastic events have malignant features and are reserved for poor prognosis (2, 4, 5). In decreasing order, the most common tumor origins and affected organ systems are mesenchymal, epithelial, and round cell, involving integumentary, hemolymphatic, digestive, endocrine, and reproductive systems, respectively (2, 4, 5).

The diagnosis of “anaplastic” giant-cell sarcoma (GCS) represents a histologic and immunohistochemical diagnostic challenge in human and veterinary medicine (6). The literature attempting a detailed immunohistochemical characterization of GCS is scarce in domestic animal species (7–9). Herein, this study reports the clinical, macroscopic, and histologic features, together with an immunohistochemical assessment, of a renal GCS in an African pygmy hedgehog with metastasis contributing to the tumor characterization and the growing knowledge of neoplasms affecting this species.

## Materials and methods

A 2-year and 7-month-old, male intact, white and black, domestic African hedgehog was presented to a private veterinary practice with a history of dry and itchy skin, ongoing lethargy, reduced fecal and urine output, anorexia, and weight loss (705–540 grams). Dermatophyte culture on skin was negative. The animal was found dead and submitted for full post-mortem examination. A standardized necropsy was performed, and representative samples from major organ systems, including skin, skeletal muscle, liver, kidney, spleen, lung, heart, adrenal gland, small intestine, large intestine, and brain, were collected and processed routinely in the histologic laboratory of the Veterinary Pathology Centre (University of Surrey). Ancillary histochemical stains, including Periodic-acid Schiff (PAS), Giemsa, Perls’, Masson Trichrome, and Von Kossa, were performed on the primary neoplasm and metastatic sites. Immunohistochemistry (IHC) was conducted on both the primary tumor and metastases for vimentin, thyroid transcription factor-1 [TTF1], Ki 67, thyroglobulin, cytokeratin (AE1/AE3, CK19, and CK20), Iba-1, S100, synaptophysin, and CD31 (Table 1) following standardized protocols (10). Internal positive controls included vascular endothelium for CD31, stromal and endothelial cells for vimentin, resident macrophages for Iba-1, Schwann cells and dendritic cells for S100, neuroendocrine cells for synaptophysin, bronchial epithelium for cytokeratins (AE1/AE3, CK19, and CK20), proliferating cells (mitotic figures) for Ki-67, respiratory epithelium for thyroid transcription factor-1 (TTF-1), and, if present, thyroid follicular cells for thyroglobulin. External positive tissue controls included canine normal-haired skin, lung, lymph node, and adrenal gland. Negative controls were produced using Primary Antibody Diluent (Leica Biosystems) instead of the primary antibody. The Bond Polymer Refine Detection Kit (Leica Biosystems) was used for visualization with hematoxylin counterstain.

**Table 1:** Primary antibodies and immunostaining protocols used in the current study.

Antibody	Source	Host	Type	Clone	Antigen retrieval	Dilution
AE1/AE3	Dako	Mouse	Monoclonal	AE1 y AE3	Citrate buffer	1:100
Vimentin	Dako	Mouse	Monoclonal	V9	Citrate buffer	1:100
TTF1	Abcam	Rabbit	Monoclonal	SP141	Tris-EDTA buffer	1:100
Thyroglobulin	Dako	Rabbit	Polyclonal	1D4	Citrate buffer	1:1500
CK20	Leica	Mouse	Monoclonal	B170	Citrate buffer	1:100
CK19	Leica	Mouse	Monoclonal	B170	Citrate buffer	1:100
Ki67	Dako	Mouse	Monoclonal	MIB-1	Citrate buffer	1:200
S100	Novacastra	Rabbit	Polyclonal	NCL-L-S100p	Citrate buffer	1:2000
IBA-1	Wako	Rabbit	Polyclonal	IBA-1	Citrate buffer	1:1000
Synaptophysin	Leica	Mouse	Monoclonal	27G12	Citrate buffer	1:100
CD31	Dako	Mouse	Monoclonal	JC70A	Citrate buffer	1:40

## Results

**Figure 1:**
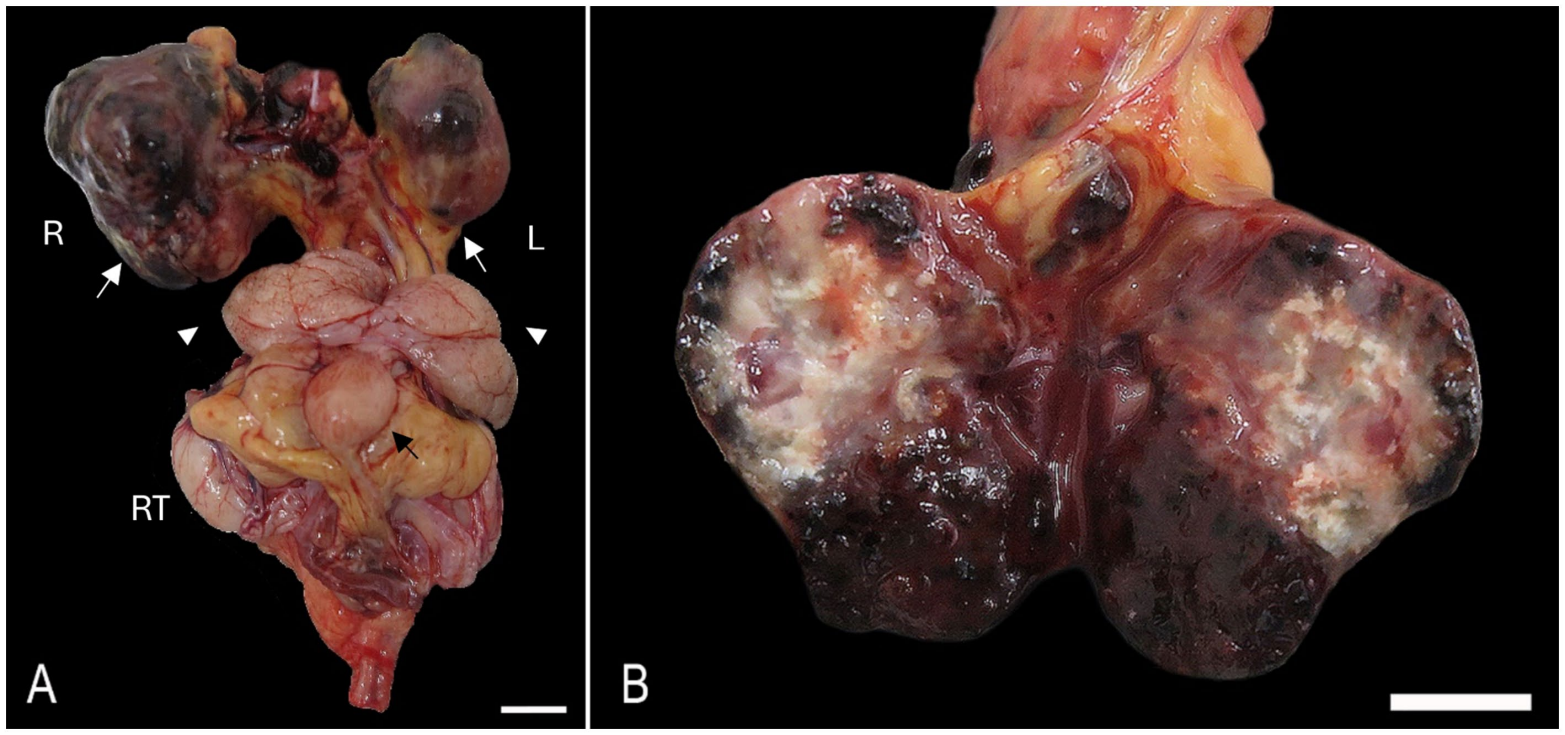
Bilateral anaplastic (giant cell) renal sarcoma in a hedgehog. **(A)** Both kidneys [white arrows, right (R) and left (L)] are expanded by poorly demarcated, irregularly round, raised, tan to dark-red nodules. The vesicular gland (white arrow heads), urinary bladder (black arrow), and right testis (RT) are visible. Bar = 1 cm. **(B)** The cut surface of the left kidney exhibits multiple colors with areas of necrosis, mineralization, and hemorrhage. Bar = 1 cm.

At necropsy, the right kidney was markedly expanded by a 2.5 × 2 × 1.5 cm, firm, multinodular, dark red to pale brown mass. The left kidney was less affected but exhibited similar findings (Figure 1A). The core of the mass was composed of multiple and coalescing blood-filled cavities intermixed with multifocal and extensive white and hard areas (dystrophic mineralization) (Figure 1B). The interrenal fat and lymph nodes, in close association with the renal artery and vein, were effaced by a focal 0.5 × 0.2 × 0.2 cm multinodular mass exhibiting identical gross features as described in the kidneys. Similar multinodular structures were present at the base of the heart and attached to the pericardial sac. The trachea was filled with moderate amounts of foam. Lung lobes were congested and edematous with occasional miliary dark red and firm areas randomly distributed throughout the pulmonary parenchyma on cut section.

**Figure 2:**
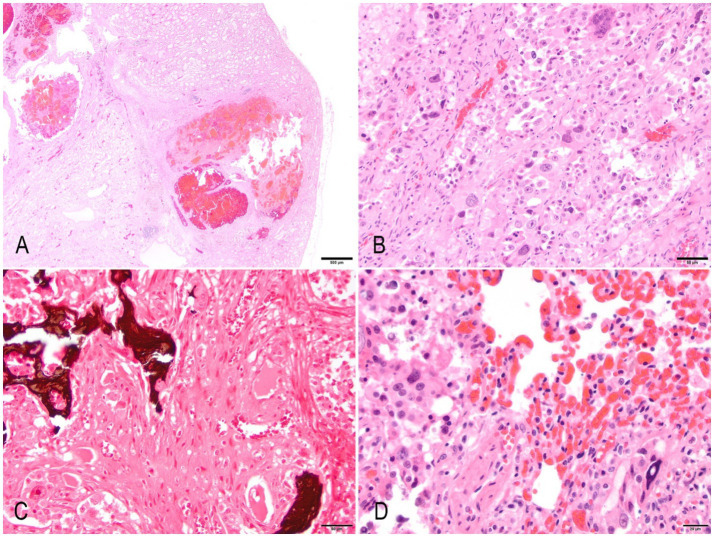
Anaplastic (giant cell) renal sarcoma in a hedgehog. **(A)** A low-power magnification image of the left kidney exhibits multifocal areas of neoplasia that efface and expand the parenchyma. The neoplasm exhibits multifocal cystic areas and hemorrhages. Hematoxylin & Eosin (HE). Bar = 500 μm. **(B)** The neoplasm is composed of spindle, pleomorphic cells that exhibit marked anisokaryosis, anisocytosis, cytomegaly, and karyomegaly. There are frequent multinucleated giant cells. Bar = 50 μm. **(C)** Multifocal areas exhibit osteoid that blends with neoplastic cells. Von Kossa. Bar = 50 μm. **(D)** Lung metastasis. Spindloid neoplastic cells infiltrate the lung parenchyma. There is diffuse marked congestion of alveolar capillary beds (HE). Bar = 20 μm.

Histologically, the cortex and medulla of the right kidney were markedly disrupted by a multilobular, expansile, moderately cellular, poorly demarcated, non-encapsulated, and infiltrative neoplasm composed of a pleomorphic mesenchymal cell population. Neoplastic cells are disposed in interwoven streams and supported by moderate fibrovascular stroma often forming large blood-filled cavities. Cells are large (60–120 μm), round to polygonal with moderately distinct cell borders, a mild to moderate amount of finely granular to vacuolated cytoplasm, a large (5–30 μm) central to paracentral nucleus with finely stippled chromatin, with an often prominent single, occasionally two, magenta nucleolus. Anisocytosis, anisokaryosis, and karyomegaly are marked. Binucleated and multinucleated giant cells were common. The mitotic count was 9 in 10 high-power fields (2.37mm^2^). Multifocal areas within the neoplasm exhibit loss of any recognizable architecture and cellular integrity with karyorrhexis and karyolysis (necrosis), numerous acicular cholesterol clefts with xanthogranulomatous reaction, and multifocal well-differentiated, eosinophilic, mineralized, and anastomosis trabeculae containing a moderate number of osteocytes (osseous metaplasia) (Figures 2A–D). The left kidney exhibited identical but less prominent histologic findings. Other microscopic findings in the kidney included moderate and multifocal lymphoplasmacytic interstitial nephritis with moderate fibrosis, frequent loss of “back-to-back” tubular arrangement, and frequent thickening of Bowman’s capsule.

The lung parenchyma was multifocally expanded and disrupted by coalescing nodules of neoplastic cells displaying similar features as previously described in the kidneys, often invading vascular structures. Multiple areas of necrosis, discrete dystrophic mineralization with occasional corpora amilacea, hemorrhages, and moderate alveolar histiocytosis were present within the neoplasm (Figure 2). The perirenal and mediastinal lymph nodes were multifocally effaced by neoplastic cells, which spread to the perinodal adipose tissue.

Secondary findings included moderate multifocal and centrilobular hepatic necrosis with marked midzonal congestion; marked splenic lymphoid depletion with abundant megakaryocytes and extramedullary hematopoiesis; mild, multifocal, chronic lymphoplasmacytic balanoposthitis with acanthosis; and mild, multifocal, perivascular lymphoplasmacytic superficial dermatitis with lymphangiectasia, oedema, reduced numbers of pilosebaceous units, and occasional superficial serocellular crusts. The presumed newly formed osteoid stained positive f von Kossa. Masson’s trichrome highlighted moderate intra-tumor proliferation of connective tissue supporting the neoplastic cells. Perls’ stain identified numerous hemosiderin-laden macrophages and multinucleated giant cells within the alveoli. Giemsa and PAS were unremarkable.

Neoplastic cells exhibited negative nuclear or cytoplasmic immunolabelling for cytokeratin (AE1/AE3, 19, 20), thyroglobulin, thyroid transcription factor 1 (TTF1), S100, synaptophysin, CD31, and Iba-1. External and internal positive controls stained appropriately in the expected different cell populations (epithelial cells, pneumocytes, nerve cells, endothelial cells, and macrophages). Vimentin and Ki-67 did not cross-react with hedgehog tissues, as evidenced by the absence of immunoreactivity in tissue elements and mitotic figures, respectively.

## Discussion

The macroscopic, histologic, and overall immunohistochemical evaluation of targeted organs in this hedgehog supported the diagnosis of renal anaplastic giant-cell sarcoma (GCS) with invasion and metastasis of the lung, mediastinal, and renal lymph nodes. The morphology and immunoprofile of the neoplastic cells warranted differential diagnoses of osteosarcoma, due to an osteoid-like matrix, and rhabdomyosarcoma, based on pleomorphism and giant cell formation. Rapidly expanding literature reveals a high prevalence of neoplastic events in captive African pygmy hedgehogs (2, 11). As expected, age increases the incidence of neoplastic events in hedgehogs, although reports in young individuals have been described (12).

In a recent retrospective study, neoplastic lesions were present in 60% of the evaluated individuals, with 76% of those being malignant (3). Commonly reported cellular origins, in decreasing order, are: mesenchymal, epithelial, and round cell. Most commonly affected systems with examples of reported neoplasms include: integumentary and soft tissue (fibrosarcoma, hemangiosarcoma, histiocytic sarcomas, squamous cell carcinoma, lymphoma, mast cell tumor, liposarcoma, sebaceous gland carcinoma, schwannoma, neurofibroma, and multicentric), hemolymphatic (lymphoma, eosinophilic, and myelogenous leukemia), reproductive (uterine adenocarcinoma, adenosarcoma, leiomyosarcoma, spindle cell sarcoma, and endometrial stromal sarcoma), mammary gland (mammary adenocarcinoma), digestive (salivary gland adenocarcinoma, colonic mucinous adenocarcinoma, gastric adenocarcinoma, hepatocellular carcinoma, and oral squamous cell carcinoma), among others (3, 11, 13, 14). Occasionally, retroviral particles have been identified within multicentric soft tissue sarcomas, and there has been speculation about the role of viruses in the development of these neoplasms (4).

The kidney was identified as the primary site based on the presence of a large, well-demarcated, expansile, and infiltrative mass replacing a substantial portion of the renal parenchyma, with disruption of normal architecture. No comparable primary lesion of similar extent or organization was observed in other organs. The distribution and progression of lesions were consistent with secondary metastatic spread rather than synchronous tumor development, supporting the exclusion of a multicentric sarcoma (13). Descriptions of primary neoplasms arising from the urinary system in the African pygmy hedgehog are scarce, encompassing adenocarcinoma, hemangiosarcoma, stromal-type nephroblastoma, and a poorly differentiated renal neoplasia (12, 15, 16).

In the former, renal tumor cells displayed negative immunolabelling against all applied antibodies. An epithelial origin (carcinoma) is discarded following negative labeling for AE1/AE3 cytokeratin. In addition, there was no immunolabelling for CK19 (urothelium) and CK20 (variably immunolabelling for urothelium/oncocytomas) (17, 18). The absence of any nuclear labelling against TTF1, together with a negative assessment against thyroglobulin, disregards a thyroid origin of the neoplasm (19). Negative immunolabelling against synaptophysin, S100, Iba-1, may exclude potential neuroendocrine, peripheral nerve sheath, and histiocytic origin of the tumor, respectively (19). Ki67 was not identified within the neoplastic cells despite a moderate mitotic count, suggesting that the Ki67 antibody may not have cross-reacted with the target antigen in this species, likely due to species-specific antigenic differences (20). Similarly, vimentin did not cross-react with hedgehog tissues, likely due to species-specific antigenic differences. The histological and immunohistochemical results support a diagnosis of renal giant-cell sarcoma.

To the author’s knowledge, this case represents the first description of an anaplastic GCS with metastasis to lungs and lymph nodes, compiling macroscopic, histologic, and extensive immunohistochemical assessment in an African pygmy hedgehog.

Interestingly, the poor body condition with marked weight loss, together with the prominent splenic lymphoid depletion and abundant extramedullary hematopoiesis, could suggest a paraneoplastic syndrome secondary to the renal neoplasm (i.e., anemia) (6). Individuals with cancer-related cachexia could present anemia, weakness, weight loss, diminished immune function, and an altered metabolism of carbohydrates, lipids, and proteins. Paraneoplastic dermatoses associated with underlying neoplasms have been described in both humans and animals, often attributed to cytokine release or metabolic disturbances (21, 22). All the criteria needed to confirm the diagnosis of a paraneoplastic syndrome could not be proved, and this is a speculative association.

This study aims to contribute to the rapidly growing literature referring to neoplasms in the African pygmy hedgehog (*Atelerix albiventris*) with the first description of a GCS arising from the urinary tract. The high prevalence of neoplasms in this species could promote further investigations to characterize and understand the behavior of tumoral cells in non-domestic species.

## Data Availability

The original contributions presented in the study are included in the article/supplementary material, further inquiries can be directed to the corresponding author.
